# The awareness, visibility and support for young carers across Europe: a Delphi study

**DOI:** 10.1186/s12913-020-05780-8

**Published:** 2020-10-07

**Authors:** Henk Herman Nap, Renske Hoefman, Nynke de Jong, Lieke Lovink, Ludo Glimmerveen, Feylyn Lewis, Sara Santini, Barbara D’Amen, Marco Socci, Licia Boccaletti, Giulia Casu, Alessandra Manattini, Rosita Brolin, Karina Sirk, Valentina Hlebec, Tatjana Rakar, Tjasa Hudobivnik, Agnes Leu, Fabian Berger, Lennart Magnusson, Elizabeth Hanson

**Affiliations:** 1 Vilans – The national Centre of Expertise for Long-term Care in the Netherlands, Churchilllaan 11, 3527 GV Utrecht, the Netherlands; 2 The Netherlands institute for Social Research (SCP), Bezuidenhoutseweg 30, 2594 AV Den Haag, the Netherlands; 3 University of Sussex, Sussex House, Falmer, Brighton, BN1 9RH United Kingdom; 4 IRCCS INRCA, Via Santa Margherita, 5, 60124 Ancona, Italy; 5 Anziani e non solo soc. coop, Sede amministrativa e legale Via Lenin, 55, 41012 Carpi, MO Italy; 6 Dipartimento di Psicologia, University of Bologna, Università di Bologna, viale Berti Pichat 5, Bologna, Italy; 7 Department of Health and Caring Sciences, Linnaeus University, 39182 Kalmar, Sweden; 8 Faculty of Social Sciences, Kardeljeva pl. 5, 1000 Ljubljana, Faculty of Health Sciences, University of Ljubljana, Zdravstvena pot. 5, 1000 Ljubljana, Slovenia; 9 Careum School of Health, Kalaidos University of Applied Sciences, Zürich, Switzerland; 10 Institute for Biomedical Ethics, University of Basel, Basel, Switzerland

**Keywords:** Informal care, Family care, Delphi study, Adolescent young carers, Young carers, Support services, European research, Cross-national research

## Abstract

**Background:**

Across Europe, young carers (YCs) and their need for support receive limited attention in the media, policy and empirical research, even though, similar to adult carers, they also provide care to ill family members. The Delphi study, a qualitative research methodology, which provides the focus for this article, had the overall aim of exploring existing successful strategies to support YCs. Compared to YCs, even less is known about adolescent young carers (AYCs), a group that is in a critical life transition phase. The study forms part of an EU Horizon 2020 funded research project on AYCs aged 15–17 years old.

**Methods:**

A two-round Delphi study was conducted with 66 experts on YCs from 10 European countries. Topics included: (i) visibility and awareness-raising of YCs at local, regional, and national levels, (ii) current interventions to support YCs, and (iii) future strategies to support YCs.

**Results:**

Experts reported a lack of visibility and awareness about YCs in general, and AYCs in particular. Although awareness is slowly increasing in most countries, with the UK ranked highest, experts acknowledged that it remains challenging to identify YCs in many countries. Furthermore, the level and type of support available for YCs differs, with most countries mainly offering support on a local level. Diverse views were expressed regarding future strategies to support YCs. Experts highlighted the importance of specific legislation to formalise the rights of YCs, and the issue of whether young people should be safeguarded from caregiving or if this should be considered part of regular family life. They also emphasised the relevance of available integrated support services for YCs, including schools, family, health and social care.

**Conclusions:**

In most European countries, there is a lack of awareness and visibility on YCs. Identification of YCs is a crucial first step and there is need for a common definition of YCs, together with greater opportunities for young adults to identify themselves as YCs.

## Background

In families where one family member has a physical or mental health problem, children or adolescents are often involved in caregiving roles [1, 2]. These young people are defined in the literature as young carers (YCs), that is: “young people under the age of 18 who provide care, assistance or support to another family member. They carry out, often on a regular basis, significant or substantial caring tasks and assume a level of responsibility that would usually be associated with an adult” [3]. These tasks are, among others, administrative and/or household tasks, personal or nursing care and/or providing company to an ill family member [4]. Besides these caring tasks, YCs often worry about their ill family member. It is not only the practical, visible tasks YCs are engaged with, but also the ‘worries in their head and in their hearts’ over the health and well-being of their family member [5].

Growing up with an ill family member is particularly recognised as a risk factor for mental health and well-being [4, 5]. Also, being a YC increases health inequalities during the life course [6–9]. It is known that YCs often experience the consequences of social exclusion, with higher absenteeism and drop-out rates from education and lower employability than their peers without an ill family member [7, 10–12].

The number of recognised YCs is relatively low yet varies per country and region [13]. It is important that YCs are identified and recognised in order to positively impact their well-being and mental health [5]. A promising way to facilitate this could be the use of technology, such as online support groups or gamified apps that could support YCs and strengthen their resilience in the transition to adulthood [14, 15]. A recent Swiss study focused on the needs of YCs for support and relief [16], however, overall there remains a dearth of knowledge about YCs’ needs and preferences for support and the ways in which (if any) they are currently being supported.

Thus, in order to address this knowledge gap, the overall goal of the current study was to gain insights into the awareness and visibility of the situation of young carers (YCs), with a specific interest in adolescent young carers (AYCs) aged 15–17 years old due to their critical life transitional phase to adulthood. The purpose was to identify their future support needs and preferences with a focus on promoting their mental health and well-being. The Delphi study described in this article forms part of a larger EU Horizon-funded research and innovation project, [17] (‘Psychosocial Support for Promoting **Me**ntal Health and **We**ll-being among Adolescent Young Carers in Europe’; ME-WE project), dedicated to strengthening the resilience of AYCs in transition to adulthood (15–17 years old) in order to impact positively on their mental health and well-being and to mitigate the negative influence of psychosocial and environmental factors in their lives [17]. The Delphi study formed part of the first phase of the project, which aimed to systematise knowledge on YCs by focusing on successful support strategies.

The aim of this article is to present and discuss the main and overall Delphi study results focusing on i) the visibility and awareness-raising of YCs on a local, regional, and national level; ii) current interventions to support YCs, and iii) future strategies to support YCs.

## Methods

To address the above core aims, a two-round Delphi study among YC experts was conducted. The Delphi method is an acknowledged qualitative research method to gather different opinions of experts, cultures and countries, and search for consensus on a topic, especially in a new field of study such as AYCs, with the possibility of diverging views [18]. A Delphi study ensures anonymous responses, which are aggregated and shared with participants after each round. Experts are allowed to adjust their answers in upcoming rounds and reflect on the results from the other participants. In this study, the goal was not to reach full consensus, but to search for consensus on certain topics and identify differences between countries in two interview rounds.

Central in round 1, were the experiences with - and knowledge on - YCs. Interviews also focused on existing strategies and programmes (if available) to improve (A)YCs’ mental health and well-being known by the expert panel. Specific attention was paid to the opinions of the panel on barriers and drivers of these existing strategies and programmes. Round 2 was performed to discuss the results from round 1 and to gather an insight into optimising programmes and developing future scenarios to best support AYCs.

### Recruitment

In total, 66 participants, i.e. ‘experts’ participated in the two-round Delphi study (see Table 1 for an overview per country). Participants were intentionally selected based upon the EU ME-WE project partners’ knowledge and professional network on YCs or related fields. All the experts had been working in the field of YCs or related fields, if not available in the country (such as youth policy), with an identifiable track record (e.g., peer-reviewed publications, organisation of events/programmes for YCs and/or young adults, development and support of care or social policies, practice: in health, social care or education). The eligibility of the experts was cross-checked by the national investigator teams. One expert from the Netherlands was not able to participate in the second round. A couple of candidates who were approached, recommended other experts (with name) more knowledgeable about the topic than themselves. These experts agreed to participate. They received an invitation for the individual interview by email, including a questionnaire in English or, if preferred, in their desired language to gather some basic characteristics, such as demographics, occupation and experience with the topic of (A)YCs, and an informed consent form agreeing to their participation and audio recordings of the interviews. The informed consent form also included a letter with information on the aim of the study and the interviews, and the applied method of the Delphi study. Furthermore, the experts received information on the project leader of the Delphi Study and the national investigators in the ME-WE-project.

**Table 1:** Descriptives of the experts per country that participated in both Delphi rounds

	Round 1 & Round 2		Main Occupational Field				
	*n*	Female n	Academia	Education	Policy	Health Care	Social Care
Italy	10	8	2	2		3	3
The Netherlands^a^	10	8	2	2	1	1	4
Slovenia^b^	9	2	1	3	1	1	2
Sweden	10	9	3		2	3	2
Switzerland^c^	10	4	1	1		5	1
United Kingdom	13	9	5	1	1	2	4
Austria	1	0	1				
Belgium	1	1			1		
Ireland	1	1			1		
Germany	1	0		1			
**Total** ***N***	**66**	**42**	**15**	**10**	**7**	**15**	**16**

^a^ 1 expert could not participate in Round 2

^b^ of 1 expert the occupational field data was missing

^c^ of 2 experts the occupational field data was missing

### Ethics

Before the start of the Delphi study, all experts received information on the aim of the project and the Delphi study and were asked to sign an informed consent form. The procedure included the assurance of full anonymity and the possibility to withdraw from the study at any stage without explanation and without consequences. All experts gave consent for participation and use of the findings for publication prior to both rounds 1 and 2 of this Delphi study.

### Interview process

The Delphi study ran over a period of 6 months in 2018. The individual interviews in both rounds were conducted via telephone, voice Microsoft Skype or face-to-face (only in Slovenia), using an interview script translated to the national language, which was cross-checked by the national investigators (see Additional files 1 and 2 for English versions of the interview scripts). Participants were interviewed by a qualified national investigator from the ME-WE project team (MA, MSc or PhD) with multiple years of experience in performing qualitative research (see Additional file 3 for the interviewers’ personal characteristics). The interviews were recorded by means of a voice recorder or a mobile application. At the start of the interview, the interviewers introduced themselves and the ME-WE-project and reminded the experts that detailed information could be found in the information letter of the informed consent form. At the start of round 1, the interviewer defined AYCs as follows: “Adolescent young carers are children who provide care for another person (normally for other family members). They often assume significant responsibility for care on a regular basis. This responsibility is something normally associated with adults. The person needing care is usually a parent. However, it may also be a sibling, a grandparent or another relative with a physical, mental or cognitive health issue.”

A semi-structured questionnaire was used in round 1 to be able to compare the results across experts, regions and nationalities, and also to ensure flexibility for individual input. The questions were pilot-tested among Dutch experts on the topic of (A)YCs. The following three main topics were selected for the open-ended questions in the first Delphi round: 1. visibility and awareness-raising of YCs on a local, regional, and national level; 2. current strategies, interventions and/or programmes to identify or support YCs (pros & cons); 3. future needs to support the well-being and health situation of YCs (see Additional file 1). These topics were selected from an academic literature review, and a grey literature search including social media. In keeping with the main target group of the ME-WE project, respondents were informed that the main focus of the study was on adolescent young carers aged 15–17 years old. Given that it was anticipated that it could prove difficult for the participants to focus solely on the age range 15–17 years, interviewees were instructed to also consider YCs attending secondary school / high school. Furthermore, if knowledge was limited, interviewees were offered the possibility to share examples on interventions for YCs aged 8–12 years. The national investigator strived to provide at least 10 min discussion time per topic. The main topics and answers were summarised at the end of the interview, followed by an informal debriefing with the participants. In this informal debriefing, the participants were asked if they had additional questions, thanked for their participation, and given information about the second round of the Delphi study. The first Delphi round took approximately 1 h per participant and varied slightly per country.

The second Delphi round took place approximately 2 months after the first round. The procedure for the second Delphi round was similar to the first and started with a summary of the previous interview, both on a national and European level. The second interview then focused on the overall summary of the most successful strategies identified to support YCs across Europe and the future needs by various end-users and stakeholders to support the well-being and health situation of YCs, and, where feasible, specifically for AYCs. The participants could reflect on these findings from the first round and adjust their own views and options. Again, the interview lasted for approximately 1 h and the participants were asked if they were willing to participate in future studies on (A)YCs.

### Data analysis

All individual interviews were transcribed in a text editor such as Microsoft Word and relevant quotes translated to English. All national investigators analysed the content and discussed the preliminary results, first with the national investigators and later, with the investigators from the other countries. The discussion was summarized by the national investigators from the Netherlands who led the Delphi study. After this, three data coders coded the data and the code tree with an initial set of broad concepts, and a legend was shared in English with the national investigators by the lead author with sufficient flexibility to share their regional and national themes. This was followed by a thematic analysis [19–21] on a national level, and the interviews were further labelled and coded by means of the qualitative data analysis software, MAXQDA of VERBI GmbH. After analysis on the national level, themes with relevant quotes were aggregated and analysed to gather insights into generic overall themes, and also on culture- or region-specific themes. An overall summary was written by the lead partner about the most successful strategies identified to support YCs, and in particular AYCs across Europe, as well as the future needs by various end-users and stakeholders to support the well-being and health situation of YCs. The summary was sent to all participants, and they were asked to read it prior to the second Delphi round (see Additional file 2). After the second Delphi round, a narrative analysis was performed on the results per country by the national investigators followed by an overall narrative analysis by the lead authors from the Netherlands.

## Results

The findings are presented first in the form of a summative table for the Delphi study round 1 interviews, followed thereafter by a narrative synthesis of the main Delphi round 1 study findings with illustrative quotes. The Delphi study round 2 findings then follow in the form of a narrative synthesis.

### First Delphi round

Table 2 below provides a comprehensive overview of the Delphi round 1 findings per country and at European level.

**Table 2:** Summary of the Delphi results from the first round per country

	Visibility and awareness raising	Strategies, interventions and programs to support AYCs	Future needs to support well-being / health situation
**United Kingdom**	- Different abilities/accessibility of formal care for YCs in different regions - On a national level an increase of awareness by television programs	- Current policy is ‘The Care Act’ and ‘The Children and Families Act’ (2014) working together to give AYCs legal right to a carers assessment on appearance of need - Well known are hundreds of young carer projects across the country (however, severe cuts in funding) - Young Carer Health Champions programme of the NHS - Child and Adolescent Mental Health Service teams (CAMHS)	- Need for general public to know about AYCs - Austerity policies have a negative impact on their situation - New legal rights for young carers in Care Act and Children and Families Act have little actual benefit.
**Sweden**	- Lack of visibility, to very low regarding AYCs - Children as next of kin is the term commonly used. - Childhood should be free from having a caring role - AYCs not directly mentioned in Swedish legislation	- Swedish Health Care Act 2010, children have a right to receive information about their parents’ illness. This means that health care professionals have a legal obligation to provide children of parent/s with mental illness, serious physical illness or disability or have unexpectedly died, with information, advice and support - People with disabilities or severe illnesses have certain rights for help and support from the community, which means that AYCs’ responsibilities for care can be reduced. - Parental support - Beardslees family intervention – when a parent suffers from mental health problems or addiction. - Group activities for families who have a member suffering from cancer, and for families in grief - Supportive groups for children/adolescents whose parent/s have a disability, mental ill-health or addiction. - Relaxation in e.g. summer camps	- Identify fragile families at an early stage and provide support they need - Make AYCs visible - Reduce stigma - Legislation needed - Digital group meetings - Have someone to listen to their story - Education about AYCs - Funding and digital solutions to provide help and support - Increase children’s knowledge of their parents’ illness - Provide opportunities for children to talk about their situation, to meet and get support - Opportunity to relax together - Society should be responsible for all care and AYCs should be relieved from caring tasks.
**Switzerland**	- Difference in coping between Swiss migrant children was mentioned. Where Swiss children hide problems because they consider them as private, migrant children find their caring role more normal - Interventions successful at schools (local level) - On a national level no visibility - Difficult to reach group (do not communicate situation to their GPs) - Research on the topic has raised awareness with some organisations	- Few local programs to support AYCs (German part offers more than the French and Italian part) - Some programs support AYCs but focus only on children of parents with mental health problems - Focus on relieving relatives (e.g., organizing summer camp) - Role of child protection service and < 18 legislative framework - Different programs have been carried out in schools to increase awareness - Few schools offering counselling to students who identify themselves as an AYC - In one Higher Education Nursing School, the topic of ‘caregivers’ and family is taught which includes young carers. Differences between Swiss children and migrants in respect to coping (migrants caring role ‘normal’)	- Schools should support AYCs to a greater extent - Increase awareness - Children under 18 should not take on board too many responsibilities - More flexibility needed in schools - Individual as well as collective intervention are needed to address different needs of AYCs and their families - The topic should be taught in the school curriculum - Professionals need to be more aware of AYCs and understand issues in order to support AYCs better - NGO’s need more funding - Whole society is responsible and need for a cultural change
**Italy**	- Lack of visibility and awareness on AYCs at all levels - A couple of examples of visibility/awareness raising (schools & hospital) - Visibility dependent on experience of teachers or medical professionals	- A couple of known interventions (support action in a school and by ANS in area of Carpi (in Northern Italy)	- Need for information and training for all health and social professionals and policy makers - Long-term multi-actor programs (ICT app) - Promote self-awareness - Ministry of Education, Welfare and Health are responsible, as schools and regional school offices - Funding (public with private and non-profit) - Many other actions that could be applied/transferred to AYCs - Need for a law on informal carers - Local authority as main actor - Role for schools and teachers in supporting AYCs (awareness raising) - Long term programs and whole-family approach
**The Netherlands**	- Low visibility and AYCs do not always recognize themselves as AYCs. - Differences in visibility between regions, municipalities are responsible for support adult caregivers and well-being of youth (struggle) - Formal policies exist on informal care, but not young carers - Schools could play an important role for increasing visibility - Welfare organizations and youth healthcare try to increase visibility	- Plays at schools and programs to support leisure activities, resilience training, support groups, etc. - Awareness programs at high schools - Guest lessons - Online platform (e.g., Sharepoint) for AYCs - Children’s Ombudsman - Activities for young carers (meet other carers) at local support centers	- AYCs should be seen as a specific group of informal carers - Focus on AYCs own strength and do not ‘problematize’ the group - Integral approach is needed. - Strive for regulation and need for having discussion on level of responsibility suited for youngsters. - Reduce stigma. - Acknowledgement of the group. - Create funding (e.g., via municipalities) for support for young carers. - Recognition of AYCs that they are AYCs - Need for specific policy and support for AYCs and putting the topic on the agenda - Need to focus on parents of children who are responsible for their care. - Need for co-creation with AYCs. - Need for integral approach (welfare, healthcare, educational and local governments that work together)
**Slovenia**	- AYCs are an overlooked subject in Slovenia and also not regulated under any law - The importance to develop a definition, emphasizing that it does not relate to short-term, but long-term care - Organizations that are in contact with young people should be responsible for detecting the problem (primarily school, physicians and organizations engaged in the field of social home care) - There is no awareness about AYC in the educational field.		- Develop an integral approach, coordinated by different ministries, with cooperation of schools and other public institutions and NGOs, connected to children and their wellbeing. - The need to build on what we have - There is no need to develop a new system, what is needed is a cooperation between existing systems and infrastructure, good prevention programmes in the community - Raising awareness and getting in contact with AYC in the social media - Need to develop awareness and destigmatisation programmes - Need to develop working relationships with the family in which AYC is living - Empower AYCs with needed information about caring and also where he/she can turn to for support - It is important that the AYC is voluntarily caring for relatives and that he/she is not under constraint - Need for early recognition (important role for schools)
**International/Europe**	- Overall, visibility is low (e.g., also in Germany). - Large differences in visibility and level of awareness between countries. - Higher numbers than one would expect. - Focus on all children, not only 15–17 years old - Schools play a role - Conference on AYCs raises awareness - Awareness raising at European Commission by – among others - Saul Becker	- Media echo of TV shows (Germany) - Brochures at schools and doctors - Events to share experiences - Website in Austria (Superhands) - Holiday activities - Carers’ card in UK - Peer groups	- Raise awareness - Early prevention (ACE ‘Adverse Childhood Experiences’ screening) - Improve skills - Look and learn from support systems for children in similar situations (parent in prison) - Ensure that children are aware of and can access their rights - Should be less inequality within and between countries - Need for support for themselves, awareness of peers - More funding and staff at schools. - There is shared responsibility (family, parents, local authorities, occupations therapists, etc.) - More visibility of AYCs in society, for example carers week - Reduce Stigma - Practical and emotional support in schools - Need for recognition - Focus on a local level - Children have rights

### Visibility and awareness-raising

In the first round, experts reported on the low visibility of YCs across Europe, including a lack of systematic studies on the subject of (A)YCs. The term YCs is not recognisable in all countries according to the experts, which can make identification challenging. Especially on a national level, experts reported that the visibility and awareness on YCs is low. Hence, when visibility and awareness is raised, this primarily takes place on a local level. Experts did report that despite a lack of visibility, awareness has slowly been increasing in recent years supported by attention in the media, such as in television shows or in newspapers. Experts argued that the majority of health and care systems across Europe still work in silos with a lack of integration. Some experts added that this also contributes to difficulties in identifying and reaching YCs because they can fall between different care or support systems/legislations.

*“We don’t want it [young adults in the role of a carer] to occur in Sweden, I would say. So, we actually don’t see, and there isn’t so much support for them [YCs], which means that they often live in a very vulnerable situation”* (Participant 7 (P7), Round 1 (R1), Sweden).

*“I think that in Switzerland there is not much visibility [on YCs] at this moment. I think that it is a topic that no one talks about. I think the people that know about this topic talk about it. But all others they don’t know that this is a topic in Switzerland because it’s invisible.”* (P4, R1, Switzerland).

### Strategies, interventions and programmes to support YCs

Experts from most countries reported that there are existing support programmes, projects and activities relevant to YCs. It is worth noting that there were differences reported within countries and between regions. The available programmes do not always target AYCs in particular, as shared by experts from Italy and Switzerland. The programmes differ in their approach by targeting individuals or groups, their duration and frequency, and demonstrated effectiveness. Experts shared a variety of strategies, interventions and programmes, such as support groups for children and adolescents with a parent or sibling with a disability or illness. Through these support groups, YCs are provided with information and realise they are not alone. Respite care is also important to support YCs according to the experts, with activities where they can relax and detach from their home situation for a while and get in contact with fellow YCs for peer-support. In addition, there are multiple initiatives in schools to raise awareness on the subject of AYCs in school plays, guest lessons or workshops. Additionally, experts explained that to follow a whole family approach, support groups for families have been set up in various countries. Finally, training programmes exist for professionals on how to identify and support AYCs.

*“We carry out psycho-educational interventions for parents and also for children if they want. We are in the preventive sphere in our case and therefore [they] have their own space of speech, they can express as well as they can listen to their parents. Our function is to improve communication within this family. And then this improves family relationships.”* (P8, R1, Italy)

Within the interventions and programmes, experts reported a focus on a number of coping strategies for YCs, such as providing them with tools to try to gain control over the situation. In addition, several experts raised the fact that YCs may often feel responsible to do what is needed and might not self-identify as a YC because they may find caring normal and may not be aware of the concept of a YC. Furthermore, according to a number of experts, YCs rather do not want to draw attention to themselves, because they do not perceive themselves to be the one in need.

### Future support to meet the needs of YCs with a focus on supporting their well-being / health situation

Experts expressed the future needs of YCs with respect to their well-being and health situation. They argued that adults and professionals need to be better trained in identifying YCs, so they can identify who and where they are, and can offer support. Experts shared that there is a need to accept the existence of YCs and reduce the stigma of caregiving. Experts shared that we should notice children who are YCs and listen to them. Further, they argued that whenever support is developed – in digital or non-digital form – it should always be developed in co-creation with YCs to fit their needs and preferences.

Some experts expressed the need for specific legislation for YCs. At the same time, they addressed the question if, and to what extent should young people be responsible for providing care tasks. Furthermore, experts stated that there should be less inequality within countries concerning access to support services. For YCs themselves, it is important that they can get in touch with fellow YCs, face to face and/or digital, according to the experts. Furthermore, schools should be more flexible towards YCs in respect to school times and deadlines. Experts reported that there is an increasing need to adopt a perspective or approach in which the whole system, as well as the family, is involved, with collaboration between stakeholders from social care, healthcare, government, and education. Experts reported that such an integrated approach is necessary so knowledge can be shared and disseminated.

*“Public and private associations must have a family-based approach to the problem, not an individual approach. You can start from one but then you have to consider all family.”* (P10, R1, Italy)

#### Second Delphi round

The synthesised findings and results from the discussions of round 2 are presented in narrative form below, according to the main identified themes from the qualitative data analysis supported by illustrative quotes.

### Visibility

In round 2, experts confirmed the results of round 1 on low, but increasing, visibility of YCs. To support the visibility of YCs across Europe, most experts agreed and expressed the need for a European NGO with structural funding independent of national budgets and for fewer inequalities within and between countries. They also mentioned a lack of recognition and knowledge among adults working with youngsters for instance, social care and schools.

According to some experts, increased visibility of YCs might also have a negative effect. Visibility means recognising YCs as a problem, which could contrast with the idea of a family where it is viewed as natural for family members to support one another, and caring roles are viewed as being private and hidden. Furthermore, experts acknowledged that sometimes YCs themselves might not want attention.

To increase visibility, Italian experts shared that some actions currently targeting other groups, such as children (not necessarily seen as carers) of parents with mental illnesses or youngsters at risk of dropping out of school early, could be positively applied to YCs. One example of this could be an app to share information about health and social services.

*“About the AYCs’ visibility, I agree that it is quite lacking, because everything is always due to the individual action, to good sense of the individual or to the upbringing that the individual has had or to personal experience […] This in regard to visibility.”* (P3, R2, Italy)

### Awareness-raising

As found in round 1 of the Delphi study, awareness is steadily increasing, according to the experts. Experts reached consensus on the differences in the level of awareness on the topic of YCs in organisations such as schools, welfare organisations and social services, with there being greater awareness in the UK, followed by Sweden, and the least awareness in Slovenia and Italy. Moreover, concerning the role of schools, it was questioned by some experts what the extent of responsibility is for schools concerning the phenomenon of YCs.

Within countries, experts noted that channels that could be used for dissemination of knowledge - and especially individual YC stories - are reports, brochures, films, social media, and mass media. Some Swedish experts reported that the YCs they know are happy to get attention, which contrasts with the results from some other countries. Some experts pointed out that campaigns only create some awareness for a short period of time, and that sustainability of interventions and awareness-raising is highly needed. They argued that long-term awareness is not necessarily guaranteed in most countries, even in countries scoring relatively high on awareness of YCs, such as the UK. Dutch experts confirmed an increasing national awareness of YCs with a considerable shift compared to the first round of interviews - for example due to a research report on young carers by the Netherlands Ombudsman for children that was officially reported in a letter to the Dutch parliament.^1^ On an international level, knowledge could be disseminated at international conferences. The information should include a definition of the term (A)YC, their life situations, YCs’ rights, their families’ rights and available support. An introduction of a national/international day for YCs was also proposed.

*“[...] films can help to make the children’s and youth’s perspective clearer, because it affects you. That’s why we usually watch films in our meetings for children’s advocates.*^2^*There are films on the Swedish Family Care Competence Centre’s website, where children and youngsters tell their stories, making it life-like and clear”* (P6, R2, Sweden).

### Identification

Experts from diverse European countries acknowledged that on a national level, they struggle with ‘formally’ identifying YCs. Screening, assessment and early identification are needed. Whenever YCs are identified - and if they are acknowledged - then formal support should be put into place, according to experts. They see the responsibility for developing programmes and strategies as primarily belonging to the state, to support and develop laws and regulations concerning YCs, and to provide them with information and additional help to relieve YCs of their caring tasks. Experts stated that without proper services in place, the identification could feel meaningless at best, and harmful at worst.

According to the Swedish experts and one expert from Ireland, identification implies acknowledgement that YCs exist and this contrasts with a strong - mainly Western - value that young adults should not take up roles reserved for parents (parentification), i.e. (un)paid work. Moreover, experts noted that we should be aware that children may be afraid that whenever they are identified, that they may be taken away from their home by social services.

With respect to responsibilities for identifying YCs, the primary responsibility is - according to some experts - on the school system, while in addition, many experts agreed that it should be routine for healthcare professionals to always ask about children and whether they have any needs when a parent is ill. Several experts agreed that the social conditions of a child should be screened when enrolling in school, i.e. that schools should act as a gatekeeper.

Furthermore, experts suggested integrated actions in which educational, social and health services should be jointly involved. However, in contrast to the advantages of involving schools in identification and support, some of the experts expressed concerns with placing too high expectations on schools due to limitations in availability, funding, time, and formal responsibilities.

*“Across all sectors, early identification and intervention for all children in need is required. Yes, so experts identified other key stakeholders and it’s got CAMHS (Child and Adolescent Mental Health Service teams) who can play a more significant role if they are trained to deliver sessions for children and their families. Additionally, educators within the school system are important stakeholders.”* (P6, R2, UK)

### Definition

Experts emphasised in the round 2 interviews that there is a need for a shared definition and terminology of YCs and AYCs across Europe, which is crucial for identifying them. However, it was acknowledged that YCs experience their caring role differently and labels can have different meanings. Swedish experts reported that to go ahead and develop functional and effective support interventions, the distinction between the terms ‘children as next of kin’ and ‘AYC’ must be defined, clarified and disseminated. Experts from Slovenia stressed that it is important to be careful not to invent the problem by forming too broad a definition of YCs. Experts stressed that we should be cautious that the term YC takes on a negative connotation and becomes a label, in particular, in research where academics try to give insights for helping policymakers to solve citizens’ problems.

*“The young carers that I’ve spoken to don’t seem to have a consistent view on what that terminology should be, so I don’t know that there will ever be a terminology that meets the needs of everyone, and everyone is satisfied with.”* (P2, R2, UK)

*“As I understand it, in Slovenia, the definition of who is and is not a young carer will, in my opinion, affect the recognition and future definitions of this problem. Therefore, it seems logical to create this definition as broad as possible [...] to acknowledge a number of situations in which young carers can find themselves in.”* (P8, R2, Slovenia)

### Support for young carers

#### Whole family approach

It was found that most experts agreed that for interventions to be successful, it is relevant to have the family involved in the intervention and work from a family perspective. In the second round, experts explained that whenever starting from a family perspective, it could open up opportunities for identifying YCs, and the roles and needs of all family members. In addition, experts argued that starting from a whole family approach makes it possible to provide concrete, practical and emotional support to all family members, thus relieving YCs. It also makes it easier to arrange follow-ups.

Experts reported that there is a need for better services for the care recipients, as well as for relief and respite for YCs. In addition to a family-oriented perspective, it is important to look beyond the family and include the broader social network, such as friends and neighbours.

*“I mean if I look at the health field that's really where we need the focus away from the individual to the family [...] force the idea that health problems always affect the whole family and not just the individual and it's the medical field’s responsibility to look at the whole family.”* (P3, R2, Switzerland)

*“A whole family approach is [...] a very good approach. And this is a tricky one but obviously we know that the earlier you receive this kind of support, then the better. Later on, there are some things about how you might pick up these families quite early. And that’s really, really important. You can’t really optimise that if it comes in too late.”* (P3, R2, UK)

#### Interventions and personalisation

During the second round, some promising examples of personalisation of interventions were reported by experts. In the UK, the voluntary sector has historically provided the most support for YCs compared to the governmental sector, which lags behind in providing support. Experts reported on flexible interventions that are tailored to different YCs’ needs that could differ for social, financial and individual conditions. From the Swedish results, to be able to explain what they need and want, experts explained that YCs first need help to reflect on their situation, their perceptions, experiences, thoughts and feelings. Some experts pointed out that support and interventions should be provided at schools. As noted earlier, they also acknowledged it was important to create flexibility for students, for example with support of a carers’ card to ensure flexibility in homework and exams. A related issue was raised by several experts - that programmes and support should run through all levels of education - from primary school to university, i.e. transition support or transitional services. This support is important due to the gap in existing transitional services.

With respect to tailored support for young carers in the welfare sector, experts underlined that YCs need access to tools and support to find useful coping strategies and help build their own resilience, such as summer camps. Experts shared and acknowledged that it is important to be aware and observant of the risks with support groups, for example, that participants in the group influence each other negatively. Furthermore, they reported that YCs also sought more holistic support, i.e., guidance on career choices, nutrition, and life management skills. Experts agreed on some limitations of interventions used in the welfare sector. These revolved around four issues: (1) interventions not matching the needs of (A)YCs, (2) good interventions that remain underused because people are not familiar with them, (3) a lack of research to substantiate the effectiveness of interventions in the welfare domain, and (4) lack of capacity or finances to arrange formal support programmes. Experts stressed that it is important not to simply focus on and create new programmes and interventions specifically for AYCs, as support for AYCs could be included in already existing interventions and programmes designed for groups such as, informal carers or children in general. As reported by UK experts, these existing programmes could be accepted as support by AYCs, since they do not specifically focus on their role as a carer and it is important that these programmes are less dependent on funding.

*“I think in some respect, it’s gotten worse more recently as a result of cuts to local authorities [in the UK] in terms of the budgets. Some areas may have had support groups for young carers in the past but have now discontinued funding for those.”* (P2, R2, UK)

#### Online support, interest in apps and co-creation

Multiple experts expressed a preference for providing online support by means of websites or mobile applications. Overall, they agreed that modern and concrete approaches are needed to raise awareness and support YCs, such as YouTube films, social media and apps. According to the experts, there is a need for an individual approach which is based on self-organisation and is easy to access by means such as an information platform or app. UK experts also pointed to digital online-based peer support to be most effective with YCs.

Experts from a variety of countries pointed out that whenever an app for YCs is built, the organisations behind the initiative also have a responsibility to exercise control through moderation and dedicated professional support, as well as structural financing for continuation of the app. Furthermore, online information about support for YCs should be directly available and not hidden via complex menus with lots of other information on care-related topics. According to UK experts, several national online support spaces in the UK have been closed because of lack of funding. With respect to online support programmes and apps, many experts agreed that the programmes should be designed in co-creation with and for YCs.

*“If we think of ‘parental support’, if you look at how it [...] the municipalities’ websites […] It’s about fifteen clicks before you get some information about this. And I think that ‘young carers’ may be twenty-five or thirty clicks away, before you can get some information about it”* (P4, R2, Sweden).

*“I absolutely agree that the programmes should be designed in cooperation with them (AYCs), so we would be able to really originate from their needs.”* (P7, R2, Slovenia)

#### Laws and regulation

A considerable number of experts reached consensus and expressed the need for laws and regulations to formalise the rights of YCs and AYCs on a national or European level. The idea of a specific law is considered positive according to some experts, to give visibility and promote the integration of interventions but, at the same time, they emphasised that it should rather not be a rigid law and that it should not become reduced to purely financial support. Furthermore, by some experts, it was questioned what the effect could be of laws and regulation on the level of responsibility placed by society on YCs.

Some experts are impressed by the laws in Sweden (Health Care Act) and the Children and Families Act of 2014 in England and Wales. However, it is relevant to note that - according to the UK experts - the current legislation has little real benefit for YCs. Overall, according to some experts, we should rather highlight the group of YCs and support them where necessary, instead of requiring some specific legislation without being able to enforce the law and provide follow-up due to a lack of funding. Like the experts in the UK, a Swedish expert pointed that although laws are reformulated, there is a risk that this will have little impact on the individual. Experts from Slovenia also emphasised that there is no need for creating new laws/legislation, as is also reported by experts from the Netherlands. Some of the Slovenian experts stressed the need to create a small body or pressure group to address the problem of YCs. Existing laws on e.g., long-term care or youth care should be sufficient to protect and support YCs where necessary. YCs and AYCs in Switzerland could be protected by the legislative framework for young persons under the age of 18 years, and according to Swiss experts, changing the legislative framework in Switzerland is extremely difficult due to the political structure. Therefore, in Switzerland it would be better to create a new national policy first.

*“Yes, you can make regulation for that. But we all know, rules only give some direction [...]. It’s the people in society who themselves make this real [...]. And look, in the Netherlands we have plenty of good regulation. But still, we see that when people interact with one another, that people get hurt or disappointed [...]. Well, regulation is insufficient. A rule is only a kind of guideline and takes the sharp edges of injustices.”* (P3, R2, the Netherlands)

#### Training, education & the role of schools

According to the experts, there is a need to increase the training and education of care and welfare professionals and to create a common knowledge base including: how to approach children, young people and parents; how to identify YCs; how to talk to YCs; how to continue once a professional has identified a YC; and available support efforts, also at schools. In addition, schools should be more involved in identifying and supporting YCs with trained personnel. As already discussed, at the same time, experts also considered the scarce time that is available among teachers. There could be training days or networks formed that meet regularly. Such education for professionals should be included in the professionals’ basic education programs. Experts suggested that training should instead be organised for all sectors (health, education, and social).

*“What are the strategies on which a school must work? First of all, create a teacher staff meeting in which professionals are involved, who are trained on all the problems of AYCs, a teacher staff meeting that shares educational management, the teaching guidelines, and then works a lot on the class group... I think that many strategies from the point of view of the school with regard to AYCs must work on the class group, which must be self-supporting, must become a team [...] and support each other according to everyone’s needs, so for me, in school you have to work now, above all, on the class group.”* (P6, R2, Italy)

## Discussion

The study is the first cross-national Delphi study on YCs, including AYCs, providing relevant insights into the visibility, awareness, interventions and future support strategies of YCs across Europe. A heterogeneous, inter-professional and geographically spread sample of 66 experts from 10 different EU countries were involved. The experts shared their views and knowledge on YCs in two interview rounds and reached consensus on the visibility and awareness-raising of YCs on a local, regional, and national level. In addition, several strategies, interventions and programmes were identified and agreed on by the experts to support YCs. Finally, experts shared their knowledge and reached consensus on future needs to support the well-being and health situation of YCs.

With respect to visibility, YCs are an invisible and neglected group in many countries and regions. Similarly to Leu and Becker [13], the Delphi study shows that there is a general lack of awareness and support for (A)YCs across nations, with varying degrees of visibility and supporting resources available depending on the country. However, despite differences among regions, visibility and awareness are increasing in most countries and there are many initiatives to support YCs on a local level, however these are less visible. Leu and Becker [13] provided a classification of countries on six levels related to awareness and policy response to young carers. According to the authors - among others - the UK is advanced at level 2, Sweden and others at level 3 (intermediate), and at level 5 are emerging countries such as Italy, the Netherlands and Switzerland. Although the present Delphi study was not intended to provide a classification, the classification level has likely shifted for some countries compared to 2017. As discussed, support for YCs in the UK is decreasing due to reduced budgets and funding, while Switzerland and the Netherlands seem to have increased media attention and have more support programmes in place on a local/regional level. It can be argued that this study provides current evidence that could feed into an updated classification in the near future to show changes in country awareness and policy responses to young carers. Leu et al. [22] also showed that, for example, in Switzerland the visibility and awareness differ between the social, healthcare and education fields, and that professionals from the health care and education sectors are more familiar with the term ‘young carers’, but feel less responsible in comparison with professionals from the social sector. The impact of awareness campaigns using television, social networking and the media can be quite large, such as in Germany or in the Netherlands.

Concerning identification, experts expressed the need for a common definition, which is currently lacking and opportunities for young adults to identify themselves as YCs. A common definition could also facilitate gathering more insights into actual numbers of AYCs in Europe and better targeting support whenever identified. However, since some YCs have difficulties in identifying themselves as YCs and vary in their experiences and care they provide, a general overall definition and concept might be challenging to construct. Nevertheless, localised or nationalised definitions can potentially support (self)identification of YCs and AYCs. The present Delphi study shows that tools to identify YCs in schools, welfare and health care are needed. Moreover, a European or international NGO for YCs could facilitate the dissemination of current knowledge on identification and support for the education, welfare and health care sectors. A European or international NGO for YCs is also, potentially, more likely to increase long-term awareness, because they are less dependent on short-term (subsidy) financial resources (in contrast to many local organisations within countries) for their YC awareness-raising activities. With respect to identification, other countries can learn from the UK, where there is already a carers’ assessment in place. Whenever YCs are identified and made visible, then society must recognise them and also acknowledge their situation as a challenge, reduce the need for young caring and provide formal support.

Providing formal support to YCs can be difficult since informal care is characterized as being provided on a voluntary basis and usually without financial compensation [23]. According to some experts, YCs should actually not be carers in the first place. However, it should be noted that YCs do exist and may be in need of support. It is likely that there will always be young people growing up in families faced with illness or disabilities, and we should provide the support they need, for example, respite care, information, social contacts, and support at school. Related to this issue is the need for specific laws, regulation, and policy on young carers. A considerable number of experts expressed that having these in place could formalise the rights of YCs on a national and/or European level. According to Joseph, Sempik, Leu & Becker [24], rights do not necessarily need to be legal rights, yet, if they are not legal rights, how strong are these rights and are they enforceable? It can be questioned if specific laws are needed for YCs and in the present Delphi study, some experts expressed that the rights for YCs are already covered in existing (non YC specific) legislation or could be included in existing legislation for social support or informal care.

Regarding interventions for YCs, rich insights were gained in the UK successes and the hundreds of (school) programmes and interventions to support YCs. However, as noted before, these initiatives are mostly based on temporary funding, so follow-up is usually lacking. With a bearish UK economy [25], the COVID-19 pandemic and Brexit in 2020, more cuts in care are expected that could reduce the support for YCs even further. YC support should rather be an integral part of health and social care, and welfare to strengthen the sustainability of support programmes and interventions. Experts addressed the need for integrated care and support for YCs, in which schools, welfare organisations and social services work closely together. Integrated care can help to potentially improve the quality of care, engage in better performance management, inter-professional teamwork, and make clear the different roles and tasks, including commitment [26]. Professionals need to be educated about YCs - their situation and what professionals can do to support them. Creating flexibility for children/students at school is essential, e.g., by means of a carers’ ID. The UK can be used as a model on how to implement a carers’ ID, yet it is unclear if such an ID will be accepted and successful in other national contexts.

The Delphi results further illustrate that overall, to support YCs, many (mostly local) interventions are running in the various EU countries. Access to interventions and programmes vary between countries, states, municipalities, and even between schools. A time, distance, culture and language independent support platform for YCs, such as an app or online platform, could overcome inequalities between regions and countries to ensure that they can receive a basic level of support [14, 15]. In fact, the development and/or provision of an online platform or app to support YCs is preferred by many of the experts who participated in the Delphi study, who also recommend that such an app be connected to available local services. An online platform can serve as an information channel with an agenda of activities in various localities. Online welfare interventions could focus on the provision of information by, for example, flyers, children’s helplines or a national information campaign. Overall, as emphasised by the experts, co-creation is key for the success of any intervention or app, and all stakeholders and end-users should be part of the co-design process [27].

From the Delphi study, recommendations can be provided based on the main findings at EU, national, and/or regional/local level for different stakeholders, i.e. scholars, policy makers, health and social practitioners, teachers and parents. Parents are the first educators of adolescents and youngsters and who also have a role in supporting young carers from a family perspective. At the research level, it would be recommended to set up parameters to identify YCs that are agreed on across the scientific community, albeit country sensitive. These should be calibrated according to the national and cultural specificities, and the services provided. Moreover, as discussed, there needs to be consensus on a common definition of “young carer” and “adolescent young carer”. This may enhance the quality of the research and the comparability of international results. It may also strengthen the evidence of the efficacy of interventions and policies to design evidence-based, psychosocial interventions and services. Research on YCs is a precondition, not only to developing comprehensive support for them, but also to identify the main gaps in the social and healthcare systems that should be addressed as a priority in order to relieve the burden on these young carers. Researchers on YCs of disabled parents also addressed a reduction in the need for young caring and this has been a long-standing call from the disability community [28–30]. The results from the present study can be used to define future research. To be effective in promoting YCs’ healthy functioning, support interventions should be evidence-based. Randomised controlled trials are currently lacking and should be encouraged in future research.

The study shows that next to scholars, full alliance is required between researchers, and health and social professionals (nurses, general practitioners, psychologists, social workers), and between the latter and the YCs. This could be accomplished by means of open listening and open dialogue with professionals and can lead to co-designed, tailored services. This cooperation may be reached by means of appropriate research methods that help the co-building of meanings and interventions, e.g. the blended learning networks (BLNs) employed within the EU ME-WE project [17]. A BLN is a group of people (i) who share a common interest, (ii) contribute with expert and/or experiential knowledge, (iii) have commitment and enthusiasm to work together to achieve (a) common goal(s), and (iv) includes key stakeholder groups. The members of a BLN together create a learning network, engage in a learning project and their meetings take place ‘face to face’ and/or electronically [31].

Concerning the education, welfare and healthcare sectors, training for teachers, health professionals and social workers is needed at local level to enhance the capability of recognising YCs, help orient YCs to the most appropriate service, and to avoid paternalism and involuntary processes of stigmatisation. It is also valuable if the education, welfare and healthcare sectors strengthen their cooperation and hence, offer more integrated care to YCs and their families. Here, it is also important to apply a family perspective and focus on the whole system, and not merely the YC or the care recipient.

To address the general lack of awareness facing YCs across Europe, as also found by Leu & Becker [13], following on from the Delphi study findings, media campaigns are recommended as a way of increasing general societal awareness that young people can be carers. At a national level, guidelines for the identification and management of YCs should be delivered and spread as much as possible within diverse sectors. Moreover, since awareness is steadily increasing, there seems momentum to set up European policy and further support NGOs such as the Eurocarers Young Carers Working Group that address the topic of YCs and AYCs and ensure continuity, without being dependent on funding as demonstrated in the UK where support programmes are steadily disappearing due to decreasing funding opportunities.

No study comes without limitations, and the main limitations of this study are related to the recruitment of the experts, the means of conducting the interviews, and the involvement of several different interviewers and research staff in the various countries, resulting in a variation in the qualitative analysis of the country-specific data. The experts were known by the ME-WE project consortium, which consists of researchers, educators and representatives from civil society - or recruited via included experts - thereby resulting in a selection bias. To address this potential bias of using convenience sampling, future studies on this topic could make a call for participation of a national or European level of expert panels which would better reflect the EU situation. Nevertheless, since the (research) field of AYCs is relatively small and even non-existent in some countries, we did manage to include 66 experts from 10 different European countries who overall reached consensus on the visibility, awareness, and needs of (A)YCs. The Delphi study focused mainly on scholars, policy makers and health service providers, and it would be supportive for the development of future support programmes to have an additional specific focus on educators and school staff. In addition, future research could extend the Delphi study with European policymakers on the topic of YCs to gain more insights into differences in policy between countries, to extract best practices and to build European policies to support AYCs.

The interviews varied in the way they were held, from telephone, voice conferencing to face-to-face interviews. It is possible that the various interview methods had an influence on the flow of the interviews and results. Telephone interviews limit visual cues resulting in a loss of contextual and nonverbal data and compromise rapport, probing, and interpretation of responses [32]. However, telephone interviews may allow respondents to feel comfortable and relatively anonymous, which is particularly relevant in Delphi studies with possibly conflicting views and opinions among experts. In addition, evidence is lacking that telephone interviews produce lower quality data [32]. Finally, multiple national investigators from the ME-WE project consortium in the six partner countries performed the interviews and first data analysis on the transcripts of the experts from the countries. The variability between the countries might have resulted in a bias between the quality of the interviews and following data analysis. Yet, all national investigators received training and specific instructions on how to perform the interviews and data analysis, including a webinar and preliminary code trees for analysis. In addition, since the interviews had to be performed in the native language of the interviewees (e.g., Dutch, Slovenian, and Italian), a relatively large group of national investigators were required for the Delphi study.

## Conclusions

In this cross-national two-round Delphi study, insight was provided into the visibility, awareness, interventions and future needs of young carers (YCs), and more specifically, adolescent young carers (AYCs) aged 15–17 years, across Europe. Sixty-six experts on YCs from Sweden, Switzerland, United Kingdom, Italy, Slovenia, the Netherlands, Austria, Belgium, Ireland, and Germany reached consensus on a number of topics. Namely, that there is a lack of visibility and awareness about YCs and hence difficulty in identifying them. Identification of YCs is crucial for providing support and a common definition of YCs and AYCs is required, together with possibilities for young people to identify themselves as carers. In this regard, practical tools are needed to aid recognition of YCs and their needs and preferences. However, identification alone is insufficient, as recognition of YCs by society as a whole is required, together with the necessary resources to secure integrated support services for and with YCs. These must address the various needs of YCs and include family, schools and the welfare and healthcare sectors. Furthermore, the level and the type of support available for YCs differs between countries, with many countries mainly offering support on a local rather than national level. Divergent views were found concerning specific legislation and needs for future support. To conclude, although there are country differences in the levels of awareness, visibility, services, and needs for support for YCs, many commonalities were observed between countries regarding challenges to accurately address the often overlooked situation of YCs in Europe.

## Supplementary information

**Additional file 1.** ME-WE interview guide of round 1 in English, The interview guide that was used during the first round of the Delphi study.

**Additional file 2.** ME-WE interview guide of round 2 used in the UK, The summary of the results from the first round; 1. General results first round (per topic), 2. Country specific results, and 3. In-depth results from the country where the interview took place (this case UK). After every section, questions where added for the experts.

**Additional file 3.** Overview of the interviewers that performed the Delphi studies, The personal characteristics are presented including their names, credentials, occupation, gender, experience and training.

## Data Availability

In accordance with the ME-WE project’s data management plan, the datasets used and/or analysed during the study are available at the end of the project from the corresponding author.

## Abbreviations

ACE: Adverse childhood experiences
AYC: Adolescent Young Carer
EU: Europe
ID: Identification
MA: Master of Arts
ME-WE project: Project on Psychosocial Support for Promoting Mental Health and Well-being among Adolescent Young Carers in Europe
MSc: Master of Science
NGO: Non-Governmental Organisation
PhD: Doctor of Philosophy
YC: Young Carer
UK: United Kingdom

## Footnotes

1 *Kinderombudsman (2018). Hoor je mij wel? - Kinderen van ouders met een ziekte, verslaving of beperking. KOM003/2018. TK (2017/2018). Brief over Kinderen van een langdurig zieke ouder Tweede Kamer, vergaderjaar 2017/2018, 30,169, nr. 70.*

2 *Many Swedish municipalities (but not all) have official children’s agents or advocates. Their main task is to ensure that the children’s perspective is considered in the community work, and to ensure that children’s needs are recognized and given attention.*
