# Supplementary material for: The awareness, visibility and support for young carers across Europe: a Delphi study
Source: BMC Health Serv Res. 2020 Oct 7;20:921. doi: 10.1186/s12913-020-05780-8 (PMC7540437; doi:10.1186/s12913-020-05780-8)
Supplement: Supplementary file 1 — Additional file 1. ME-WE interview guide of round 1 in English, The interview guide that was used during the first round of the Delphi study. [file 12913_2020_5780_MOESM1_ESM.pdf]

ME-WE Delphi Study

This project has received funding from the European Union's Horizon 2020 research and innovation programme under grant agreement No 754702"

Delphi script round 1

0-5 minutes: Introduction

"Hi, this is XYZ. I really appreciate your participation in the ME-WE Delphi study. You received the information sheet by email and already filled in the general questionnaire and informed consent. In the information sheet it was mentioned that you could ask questions before the actual interview by email or telephone. Do you have any additional questions or questions that came up now? Was everything clear?

Remember that the upcoming interview is not a test or experiment. Your personal opinion is highly important to us and understand that everything you say is analysed anonymously. The interview will last approx. 45 minutes and we will discuss 3 topics and I will provide you with a summary at the end and remind you about our following interview (second round). I will make notes during the interview and the interview is recorded and later transcribed for further analysis. As stated in the information sheet, the audio file is stored on a secured server without reference to you and will be destroyed after 10 years. Let us start with the interview and feel free to ask if something is unclear…"

5-15 minutes: Topic 1

"As you know, today we will be talking about your experiences and knowledge of Adolescent Young Carers and/or related fields. Adolescent Young Carers (AYCs) are aged between 15-17 years. Young Carers (YCs) are defined as "'young carers' are children who provide care for another person (normally for other family members). They often assume significant responsibility for care on a regular basis. This responsibility is something normally associated with adults. The person needing care is usually a parent. However, it may also be a sibling, a grandparent or another relative with a physical, mental or cognitive health issue."

The first topic you **can share information about is the visibility of AYCs on a local, regional, and national level."**

Possible sub- / probing questions: "Do you have information about the percentage of young adults that provide informal care? "; "Which organization(s) are responsible for identifying Adult Young Carers"; "How are the identified?"

[SEE BELOW WHENEVER THE ANSWER IS THERE IS NO VISIBILITY AND AWARENESS RAISING] Possible sub- / probing questions: "Do you have information about the percentage of young adults that provide informal care? "; "Which organization(s) are responsible for identifying Adolescent Young Carers (if available in country)"; "How are they identified?"

Additional questions Group 3 (with no knowledge on YCs, possibly some of group 2)

- *"We know that Young Carers are less visible on a local, regional, and national level in Italy/Slovenia/?. Maybe you can share information on a broader level and think about:"*
- *"Who could be a Young Carer in Italy/Slovenia/??*
- *Which characteristics (e.g. family composition, economic status, educational level, urban/rural environment, etc). may have a Young Carer in Italy/Slovenia/??"*
- *"Which type of relative do you think a young person could care for in Italy/Slovenia??"*

Sweden: first focus on children as next of kin, then AYCs.

15-30 minutes: Topic 2

First & Second Group

"The second topic **you can share information about are the current strategies, interventions and/or programmes to support (A)YCs** [IF NO EXPERTISE, THEN ABOUT YOUNG ADOLESCENTS] and enhance their health and mental well-being?

Furthermore, per strategy mention the specific aims of the intervention (e.g., reducing school drop outs, social inclusion, leisure), and try to mention 2-3 aspects that worked well and 2-3 aspects that did not work well (pros & cons)".

Possible sub- / probing questions: " What are the strengths and weaknesses of this programme in supporting (A)YCs?"; "How could you migitate the negative influences of psycho social and environmental factors (resilience)?"Could you share some additional background on theories that strengthen/enhance coping strategies"; "Which organizations are involved in this local/regional/national programme?"

Additional questions Group 3 (with no knowledge on YCs and/or with knowledge on development/coping theories, possibly some of group 2)

- *"Could you share some background on theories about developmental stages of children and adolescents that could be applied to the Young Carers in Italy/Slovenia/??"*
- *"What do you think would work" OR " if he/she can think about intervention not specifically targeting our target group that would work well"?*

30-45 minutes: Topic 3

"For the final topic you can share your vision or forecast on future needs to support the well-being and health situation of (A)YCs.

When known, you can think about and cluster the needs by the needs of (A)YCs, schools, but also informal carers, care professionals and/or policy makers." Possible sub- / probing questions: "How can we optimally support AYCs?"; "From theory or practice, what key elements should be included in a support programme or application that support AYCs?"; "What format or means would work to reach out to this group of AYCs?"; "Who is/ should be

responsible?"; "Which network/stakeholders should be involved (e.g. social and health services, public authorities, schools, NGOs, etc.), and at which level (e.g. national, regional, local)?"; "How to fund the suggested supporting programme"?

45-50 minutes: Wrap-up

"I will summarize now the main/key points that were discussed for topic 1, 2 & 3. Did you have anything to add or tell? Which issues do you consider are important or were most important?" "I thank you for your participation in this interview. Your input is highly appreciated. We will transcribe the interview and analyze the main findings. Before the second interview that we could plan now (or via a Doodle), you will receive the preliminary findings from all interviews. In the second interview we will further discuss the results from your first interview, mainly on topic 2 & 3, in light of the results from the other interviews. Thanks again and talk to you on XYZ"
