# Supplementary material for: The awareness, visibility and support for young carers across Europe: a Delphi study
Source: BMC Health Serv Res. 2020 Oct 7;20:921. doi: 10.1186/s12913-020-05780-8 (PMC7540437; doi:10.1186/s12913-020-05780-8)
Supplement: Supplementary file 2 — Additional file 2. ME-WE interview guide of round 2 used in the UK, The summary of the results from the first round; 1. General results first round (per topic), 2. Country specific results, and 3. In-depth results from the country where the interview took place (this case UK). After every section, questions where added for the experts. [file 12913_2020_5780_MOESM2_ESM.pdf]

ME-WE Delphi Study

Expert Interviews second round v1.0

This project has received funding from the European Union's Horizon 2020 research and innovation programme under grant agreement No 754702"

Dear Expert:

- Please read the results and prepare answers for the questions after every section. These questions will be raised by the interviewer during the interview
- First, we present general findings (first part), then the summaries of different countries (second part), and then in detail the results from the United Kingdom.

Date: 16-08-2018

1. General results first round (5-10 minutes)

Visibility and awareness raising

- Overall there is a low visibility of young carers in Europe
- The term young carers is not recognizable in all the country's, this also troubles identification.
- When visibility and awareness is raising this mainly takes place on a local level
- In some countries the region you live in depends on formal support is available for (A)YCs
- Young carers are hard to identify and reach also because they can fall in between different care or support systems/legislations
- There is a lack of systematic studies on the subject of young carers
- Although there is a lack of visibility, awareness is slowly increasing in the recent years
- Attention in the media (television/newspapers) leads to a slow increase of visibility and awareness
- There is key role addressed for GPs and teachers (education) in the identification of (A)YC, for example by a carers assessment (UK)
- In most countries activities intended for young carers are initiated

Strategies, interventions and programs to support (A)YCs

- In most countries there are programs / projects / activities for young carers
- These programs don't always aim for young carers in particular (Italy/Switzerland)
- Activities differ in approach (individual/group), duration (once/regularly) and demonstrated effectiveness
- Examples of strategies, interventions and programs are:
    - Support groups for children/adolescents with a parent or sibling with a disability or illness where YCs are provided with information and realize they are not alone
    - Attention for the subject of young carers in schools (school plays / guest lessons / workshops)
    - Activities in which YCs can relax and detach from their home situations for a while and get in contact with fellow young carers for peer-support
    - Sometimes the programs are a combination of the above-mentioned programs
    - Support groups for families (a family centred approach)
    - Trainings for professionals

- Coping strategies of young carers are:
  - By taking/providing care YC try to gain control over the situation
  - They feel responsible to do what's in their power
  - YCs might ignore their role as young carer, they find it normal / don't know better
  - YCs don't like to draw attention on them (they are not the one in need)

Future needs to support well-being / health situation

- The expert need to adopt a perspective / an approach in which the whole system / family is involved
- There is a need for collaboration between stakeholders (education/welfare/healthcare/authorities), an integral approach in which knowledge is disseminated
- Support should always be developed in co-creation with YCs to fit their needs
- There is a need to accept the existence of (A)YCs and reduce the stigma
- There is a need for legislation specific for the group of young carers
- Adults/professionals need to be more trained in identifying young carers
- The opportunity for flexibility in schools for the group of young carers
- Less inequality within countries for the access to support
- Noticing children who are young carers and listening to them (eye and ear)
- The ability to get in touch with fellow young carers (face to face / digital)

Questions for Expert:

- *Could you give a reflection on these main findings?*
- *Which elements are remarkable? Could you give me a reflection on them?*

2. Country specific results (15 minutes)

Visibility and awareness raising

- United Kingdom
  - Different abilities for YC formal care in different regions
  - On a national level an increase of awareness by television programs

- Sweden
  - Lack of visibility, to very low.
  - Children as next of kin has been used.
  - Childhood should be free from having a caring role.
  - Not mentioned in Swedish legislation

- Switzerland
  - Difference in coping between Swiss migrant children was mentioned. Where Swiss children hide problems because they consider them as private, migrant children find their caring role more normal.
  - Interventions successful at schools (local level)
  - On a national level no visibility
  - Difficult to reach group (do not communicate situation to their GPs)

- Italy
- Lack of visibility and awareness on AYCs at all levels.
- Couple of examples of visibility/awareness raising (schools & hospital)
- Visibility dependent on experience of teachers or medical professionals.

- The Netherlands
- Low visibility and YCs do not always recognize them as YCs.
- Differences in visibility between regions, municipalities are responsible (struggle)
- Formal policies exist on informal care, but not young carers
- Schools could play important role for visibility
- Welfare organizations and youth healthcare try to raise visibility.
- Need for integral approach (welfare, healthcare, educational and local governments that work together)

- Slovenia
- YCs are an overlooked subject in Slovenia and also not regulated under any law
- The importance to develop a definition, emphasizing that it does not relate to short-term, but long-term care.
- Organizations that are in contact with young people should be responsible for detecting the problem (primarily school, physicians and also organizations engaged in the field of social home care)
- Raising awareness and getting in contact with YC in the social media
- There is no awareness about YC in the educational field.
- There is no need to develop a new system, what is needed is a cooperation between existing systems and infrastructure, good prevention programmes in the community

- Europe
- Overall, visibility is low (e.g., also in Germany).
- Large differences in visibility and level of awareness between countries.
- Higher numbers than one would expect.
- Focus on all children, not only 15-17 years old
- Schools play a role.
- Conference on AYCs raises awareness.
- Afraid of peers and bullying (e.g., Germany)

Strategies, interventions and programs to support (A)YCs

- United Kingdom
- Current policy is 'The Care Act' and 'The Children and Families Act' (2014) working together to give YCs legal right to a carers assessment on appearance of need
- Well known are hundreds of young carer project across the country (however, severe cuts in funding)

- Sweden
- Swedish Health Care Act 2010, children have right to receive info. about their parents' illness
- People with disabilities or severe illnesses have certain rights for help, for YCs this could possibly be arranged.
- Increase child's knowledge of their parents' illness
- Provide opportunities for children to talk about their situation, to meet and get support.

- Opportunity to relax together.

❖ <u>Switzerland</u>
- Few programs to support AYCs
- Focus on relieving relatives (e.g., organizing summer camp)
- Role of child protection service and <18 legislative framework
- Different programs have been carried out in schools to increase awareness
- Differences between Swiss children and migrants in respect to coping (migrants caring role 'normal')

❖ <u>Italy</u>
- Couple of known interventions
- Many other actions that could be applied/transferred to AYCs:
- ICT app to share info. about health and social services, workshops, alternative school programs, assistance support, whole family approach, etc.
- Need for a law on informal carers.
- Local authority as main actor.
- Role for schools and teachers in supporting AYCs (awareness raising).
- Long term programs and whole-family approach.

❖ <u>The Netherlands</u>
- Need for specific policy and support for YCs by scheduling the subject.
- Plays at schools and programs to support leisure activities, resilience training, support groups, etc.
- Recognition of YCs that they are YCs
- Need to focus on parents of children who are responsible for their care.
- Need for co-creation with YCs.

❖ <u>Slovenia</u>
- Different strategies and interventions mentioned: developing awareness and stigmatisation programmes / developing working relationships with the family in which YC is living / empower YC with needed information about caring and also where he/she can turn to for support
- It is important that the YC is voluntarily caring for relatives and that he/she is not under constraint.
- One of the important strategies is also early recognition (Important role for schools).

❖ <u>Europe</u>
- Need for recognition
- Focus on a local level.
- Children have rights

Future needs to support well-being / health situation

❖ <u>United Kingdom</u>
- Need for general public to know about AYCs
- Austerity policies have a negative impact on their situation.
- New legal rights for young carers in Care Act and Children and Families Act have little actual benefit.

❖ <u>Sweden</u>

- Identify fragile families at an early stage and provide support they need
- Make AYCs visible
- Reduce stigma
- Legislation needed
- Digital group meetings
- Have someone to listen to their story
- Education about AYCs
- Funding and digital solutions to provide help and support

❖ Switzerland
- Schools should support AYCs to a greater extent
- Increase awareness
- Children under 18 should not take too many responsibilities
- More flexibility needed in schools
- Whole society is responsible and need for a cultural change

❖ Italy
- Need for information and training for all professionals and policy makers and also teachers
- Long-term multi-actor programs (ICT app)
- Promote self-awareness.
- Ministry of Education, Welfare and Health are responsible, as schools and regional school offices.
- Funding (public with private and non-profit).

❖ The Netherlands
- YCs should be seen as a specific group of informal carers
- Focus on YCs own strength and do not 'problematize' the group
- Integral approach is needed.
- Strive for regulation.
- Reduce stigma.
- Acknowledgement of the group.
- Create funding (e.g., via municipalities) for young carers.

❖ Slovenia
- Develop an integral approach, coordinated by different ministries, with cooperation of schools and other public institutions and NGOs, connected to children and their wellbeing.
- The need to build on what we have

❖ Europe
- Raise awareness
- Early prevention (ACE 'Adverse Childhood Experiences' screening)
- Improve skills
- Look and learn from support systems for children in similar situations (parent in prison)
- Support the person
- Ensure that children are aware of and can access their rights
- Should be less inequality within and between countries
- Need for support for themselves, awareness of peers
- More funding and staff at schools.
- There is shared responsibility (family, parents, local authorities, occupations therapists, etc.)
- More visibility of AYCs in society, for example carers week.
- Reduce Stigma

- Practical and emotional support in schools
- Higher reputation -> informal care more visible

Questions for Expert:

- *Could you give a reflection on these findings per country?*
- *Which elements are remarkable? Could you give me a reflection on them?*
- *Do you identify similarities with the situation in your own country?*
- *What type of awareness and support programs might be suitable for your own country?*
- *What is needed on a global level?*

3. In-depth results from the United Kingdom (30 minutes – 10 on each topic)

1. Visibility and awareness raising

Main findings:
Research by organizations such as Carers Trust have led to the increased visibility of young carers, coupled with the passage of the recent Care Act and Children and Families Act in England (P4). The Children in Need programme in the media, coupled with more press attention (e.g., "DIY SOS" project) from celebrities with a background of young caring has helped to increase the visibility of young carers (P6; P8). A focus on young adult carers has developed because of our understanding of the importance of the transition from childhood to adulthood, however we still do not know enough about the actual numbers of young adult carers (P3; P5). It can also be difficult to define young carers and thus a "harder checklist style definition" is needed (P4). The visibility of young carers may be further hindered by a lack of advertisement in public spaces about young caring; one expert remarked that if it was not for his involvement in professional research with young carers, he has never seen a notice about young carers in a public space for the general public (P4). Notably, most children in the United Kingdom would not have had contact with a young carers service (P4). In Northern Ireland specifically, the crisis in government (devolution and its recent collapse) has played a significant role into the lack of profile-raising of young carers, as young carers simply have not been seen as a political priority (P10).

The definition of young caring defined by researchers and academics from the 1980s and 1990s has caused people to focus predominately on young people caring for parents with a physical condition or disability, rather than those young people caring for parents with mental illness. Other experts pointed to other problems with the current conceptualization of young carers, specifically with the term "adolescent young carers". It was felt that the introduction of the term was unnecessary because young people aged 15-17 years old are already included into the typical definition of "young carers" because they are legally classified as "children" (P3). Furthermore, increased categorization of young carers is unhelpful because terms such as "adolescent young carers" are not recognized in the current legislation affecting young carers (P3). What is needed is to continue to remind society of the requirements and duties already in place to recognize and support children with caring responsibilities across the health, social care, and educational sectors, particularly in light of the "Children in Need" legislation (P3). In light of the Children in Need

legislation, one expert noted that adolescent young carers may in actuality be hidden within the classification of "Children In Need" and this can serve to complicate the ability to draw out hard statistics on the numbers of young carers (P4).

There remains gaps in understanding and awareness of adolescent young carers within the educational sector; on an individual level, some teachers may feel that identifying and supporting young carers is not their responsibility, rather, it is the responsibility of social welfare (P4).

In P1's view, the Nordic countries (namely, Norway, Sweden, Denmark, Finland, and Iceland) have done particularly well in acknowledging the children of parents with mental illness (COPMI), and comparably better than the United Kingdom. P1 believes that there is an avoidance to consider the impacts of parental mental illness on a children's mind in the United Kingdom. P1 believes this is due to the fear of social service involvement and the child's removal from the parental home. Thus, P1 asserts that children caring for parents with mental illness are generally overlooked when considering the young caring experience in the United Kingdom. However, Scotland may be more forward-thinking than England in this regard (P1). P1 points to the definition of young caring promoted by early young carers researchers' did not take into account children significantly affected by parental mental illness as "young carers", and this likely influenced the hidden position of COPMI in the discussion of young carers.

P2 found it critical to remember that adolescent young carers should be firstly considered by their developmental stage and cognitive and emotional stage of development, rather than their biological age. She preferred this way of thinking because it is often thought that adolescent young carers are of the age to do "legally" and "appropriately" perform certain caring tasks, yet, their developmental maturity can vary widely.

Regarding visibility, P2 believes that there is a particular pervasive stigma for adolescent young carers because of their age; there persists an societal idea that adolescent young carers are "not working hard enough, not doing well at exams, and not working hard enough in school". This stereotype is connected to wider societal beliefs about youths and youth culture. Similar to P1, P2 believes that there is more stigma for young people caring for those with mental illness or addictions in comparison to those caring for physical disabilities. She believes that those caring for individuals with physical disabilities can receive a "hero" status, yet those caring for family members with addictions or mental illnesses do not receive that same "branding".

In Scotland, the recent passage of the new Carers Act has affected the visibility of young carers (P2). More broadly, there may be an growing awareness of the kinds of activities that young carers do, but there remains a gap in understanding about the complexities and diversity in young caring experiences (P2). Within the Scottish school system, most teachers have a reasonable awareness of young carers but do not know how to put that awareness into practice (P7). In Scottish universities, the development of a Carers Recognition Award has been helpful in helping universities identify young adult carers, but it is understood that those young people not in education remain hidden (P7). In addition to the passage of the Carers Act in Scotland, awareness of young carers in Scotland may be growing because of the history of political campaigning, coupled with the visibility of politicians speaking up about their own caring experiences, and finally the appearance of young carers in popular Hollywood movies such as the *Hunger Games* (P2). Specifically, P2 points to the level of heightened awareness in Edinburgh due to the involvement of the local young carers project into schools and its connected publicity.

2. Strategies, interventions and programs to support (A)YC

Main findings:

Adolescent young carers are identified through children's social services and that contact may lead to involvement with the over 300 young carer services (called "young carers projects") in the United Kingdom. Formal support for young carers appears to be concentrated in England, as there are not as many young carers projects in remote areas (P3). Young carers could also be referred to formal services through adult mental health services, if there is an "enlightened mental health team and an enlightened mental health consultant" (P1). Still others are referred by voluntary bodies and by schools (P1; P5). In schools in England, the National Young Carers in Schools programme includes resources for schools such as posters and other information for young people to self-identify as carers, whilst also allowing them to connect with a designated person in their school to speak to about their caring role (P5). The ability to access a young carers service may be less defined by rural or urban localities, and more dependent upon the allotment of funding (P3). Other hidden groups with low service access include Black and Minority Ethnic young carers and LGBT young carers (P7, P8). In Northern Ireland, professionals point to their unique situation of children caring for parents and other family members involved in intra-country armed conflict (The Troubles) (P10). How much funding a local authority receives for young carers and whether there is consistent services depends upon funding (P3; P10). Because of budget cuts, some young carers projects are not able to offer a transitional young adult carers service (P9). Young carers projects are indeed useful because of the way they can offer family support and consider the needs of the entire family (P3). Young carers projects also provide the opportunity to talk amongst other young caring peers and talk to adults who are knowledgeable about their family situation. The young carer project can also direct a young person to other avenues of support, respite care, or help facilitate communication between social care and schools (P4; P9). One expert pointed to the need for young carers services to have better "exit strategies" in place for when young carers transition into adulthood (P3).

Currently the NHS England runs a programme called the "Young Carer Health Champions programme" that involves a group of young carers working with the NHS for a year term (P6). It was suggested that GPs and GP attached nurses should play a more significant role in the identification of young carers, yet the overwhelming view is that they currently do not (P1). School nurses are ideally placed to identify young carers due to their close contact with children; however P1 points to the devolution of the school nursing services to local authorities as the reason for their decreased involvement in identification. Historically, carers were not a central focus in health training, and therefore even the concept of "carers" was absent from health professional language (P6). Furthermore, it was asserted that health care professionals are frightened to ask more questions leading to the identification of young carers because they feel incompetent in regards to how to direct young carers to services (P6). It was also thought that the lack of a "solid" duty (in reference to the Care Act and Children and Families Act, health professionals have a "duty of cooperation") for health professionals to engage with carers was one reason for the disengagement with young carers (P6).

There is also a need for more detailed training for professionals in the complexities of young caring experiences, family dynamics, and an understanding that young carers need ongoing conversations about their caring role (P2). Part of service delivery on building boundary-setting for young carers within their families (P2). One example of best practice shared from Scotland includes the ongoing young carers forum in Edinburgh, in which young carers led the forum with city commissioners and project workers present, providing an opportunity for them to share their views (P2). The young carers forum demonstrates the success of empowerment and skills building for adolescent young carers, as they have listed their participation in the forum on their CV and for college admission (P2). Additionally, Scotland has seen the development of a National Strategy that identified young adult carers as a distinct group (P7). At the Edinburgh Young Carers Project, there appears to be a growing acknowledgement of the specific needs of LGBT adolescent young carers evident by the continued engagement of LGBT-oriented workshops during young carers group and respite activities. This project has also seen an uptick in transgender adolescent young carers identifying themselves, which P2 believes is characteristic across Scotland.

Child and adolescent psychiatrists also should play a role in identifying young carers, however, as they currently work in Child and Adolescent Mental Health Service teams (CAMHS) which are “grossly underfunded and grossly understaffed” and at a “state of crisis in the child and adolescent mental health” (P1). This is why P1 believes that child and adolescent psychiatrists can only work to support other professionals in identifying young carers. The issues of understaffing and underfunding continue in adult mental health services and therefore the transition period for young carers aging into adulthood is also fraught with troubles (P1). Adult mental health services are “very threadbare, understaffed, and over-pressed”, and further complicating matters, adult mental health services do not typically think of the social context of the family and often claim that they do not know how to talk to children (P1). P1 calls this a “cultural problem” and has been involved in the campaign to create change in this area.

3. Views on the support needed for (A)YCs

Main findings:

Within the educational system, mentors and tutors with a good understanding of the life of young carers would be advantageous (P4).

Young carers need guidance and information in regards to their own health care needs, and health professionals are ideally placed to provide this (P6).

One expert pointed to the need to support entire families, citing high levels of unemployment and inequality facing families in Britain (P3). P5 asserted that professionals are more likely to find young carers in families living in poverty. A “sympathetic” government that is “not consistently hitting the hardest, the poorest, and most vulnerable people” is also needed (P3).

In regards to young carers’ own mental health needs, there is a need for the intervention of mental health professionals, especially in light of the dissolution of formal support services because of funding (P7).

Young people want a proper understanding of the illness affecting their parent, they want an advocate, and they want to know they are not alone and be with other young people who have similar experiences (P1). P1 points to the model used by the organization Kidstime (a formal support service for COPMI), which uses drama, awareness work in schools, separate parent and child groups, and joint groups on a monthly basis with up to 10 to 20 families. The model espoused by Kidstime helps the parents and children communicate with each other and discover a joint understanding of mental illness which they both accept and understand. P1 emphasizes such dedicated groups are critically important for children with parents affected by mental illness as a “typical” young carers group may not fully address their unique needs and experiences. P1 believes that schools have a tendency to send young carers off to a school counsellor (if there is one) as the first course of action, when in actuality, some children find a discussion of their feelings to be quite distressing as it can make them feel incompetent. P1 prefers an approach that helps a child *think* about their experience, rather than about their feelings. Young carers groups may be beneficial for children affected by parental mental illness, however it can also be stigmatizing if the group only has children with parents with physical disabilities (P1).

In regards to connection of identification and receipt of formal support services, P3 asserts that terminology must refer to adolescent young carers as “children”. P2 emphasizes that the growing identification of young carers is helpful, however, without proper services in place, the identification can feel meaningless at best, and harmful at worse. Immediate referral to a young carers service may *not* be what a young carer needs, particularly at the first instance of a parental health diagnosis (P2). Rather, ongoing discussion on a young person’s unique needs is recommended (P2). There is also the possibility that receipt of a formal supportive young carers service could enforce dependency on the service, rather than fostering empowerment and self-advocacy (P2).

4. Future needs to support the well-being and health situation of (A)YCs

Main findings:

More thought must be given to considering *why* children become young carers, as well as whether it is ethical to support a young carer in continuing their caring role (P4).

The implementation of Universal Credit means that more work must be done on young carers taking on responsibility for financial and money management within their families, in addition to their other caring responsibilities (P5; P6).

To address the fear of families self-referring themselves to social care services, local charters/covenants providing security to vulnerable families would be helpful (P4).

Employers need to be aware of adolescent young carers and have support policies in place to make the workplace environment friendlier for young carers (P7).

Children of parents affected by mental illness should have a lead member of staff trained to work with COPMI and that individual should understand when to contact suspicious and vulnerable families, how to connect the child with their parent, and have a responsibility for engaging other schools in supporting other children with parental mental illness (P1). Ideally, families should be positioned to receive the services of Kidstime if they choose. If children prefer not to use Kidstime, they should have contact with a social worker who was a dedicated 'Children in Need' for parental mental illness professional. The government should acknowledge that COPMI exist and fund social care and health services particularly at the children's level. The government should also integrate Children's Mental Health Services and Adult Mental Health Services (P1). Finally, the impacts of parental mental illness on children's minds must also be centered and addressed.

More research is needed on children caring for parents of substance misuse, and such children must be included into the definitions of young caring (P3).

Experts identified other key stakeholders who are not currently involved in the work to support young carers, such as GPs and primary care services (P3). CAMHS can play a more significant role if they are trained to deliver sessions for children and their families (P1; P4). Additionally, educators within the school system are important stakeholders. Across all sectors, early identification and intervention for all children in need is required (P3).

The period of austerity and the cuts to social care services have meant that young carers support services are intaking young people at severe crisis levels, for example, P2 points to young carers joining the service who are actively suicidal and the Mental Health Agency reports a several months long waiting list. P9

reports that he has seen an observable uptick in adolescent young carers engaging in self-harming behaviors, particularly female young carers. Furthermore, P2 affirms that the strain on social services in Scotland is so significant that their young carers service is receiving intakes from young people who are not actually young carers; this was also corroborated by P3 in England. For this reason, P2 believes that all young people who have a ill or disabled family member need a professional to assess their needs, even if they may not be providing care for their family members.

Professionals should better inform families of the benefits they are entitled to, and especially, young carers must be made aware of their rights to assessments of their needs (P5).

One expert felt that whilst the recent amendments to legislation giving further rights to young carers has been beneficial, there is also a sentiment that all has been accomplished for young carers and nothing further must be done (P3). This expert alluded to the dissolution of the National Young Carers Coalition and their view that national NGOs such as Carers Trust and the Children's Society do not appear to be leading on work for young carers as strongly as those organizations had in the past (P3).

Finally, whilst it was thought that Brexit will have disastrous effects for families across Britain, it was not thought that young carers will feel the effects as hard as other groups of vulnerable people (P3).

Questions for Expert:

- *Could you give a short reflection on these findings for the United Kingdom for 1. Visibility, 2. Strategies, and 3. Support needed and 4. Future scoping -> Overall consensus summary.*
- *Could you change, update and comment on findings that are in conflict with your experiences/expertise on AYCs in the United Kingdom -> Information that is incorrect for your country.*
